# Glucagon-like Peptide-1 Receptor Agonists: A New Frontier in Treating Alcohol Use Disorder

**DOI:** 10.3390/brainsci15070702

**Published:** 2025-06-29

**Authors:** Tyler S. Oesterle, Ming-Fen Ho

**Affiliations:** 1 Department of Psychiatry and Psychology, Mayo Clinic, Rochester, MN 55905, USA; 2 Department of Molecular Pharmacology and Experimental Therapeutics, Mayo Clinic, Rochester, MN 55905, USA

**Keywords:** GLP-1, GLP-1RA, AUD, addiction, substance use disorder

## Abstract

Background/Objectives: Glucagon-like peptide-1 receptor agonists (GLP-1RAs), which were originally developed for managing type 2 diabetes by enhancing insulin secretion and reducing appetite, have emerged as promising candidates in alcohol use disorder (AUD). These medications offer a dual mechanism of action that aligns with the multifaceted nature of addiction by targeting both peripheral metabolic and central reward pathways. This review focused on the current clinical trials and real-world evidence regarding the effects of GLP-1RAs as novel therapeutics for AUD. We also discussed early but encouraging results from clinical trials in AUD, observational and real-world evidence, safety profiles, psychiatric considerations, and future directions leading beyond GLP-1RAs. Methods: A comprehensive English-language literature search was conducted per PRISMA guidelines across PubMed, Medline, Google Scholar, Web of Science, and trial registries. Using targeted keywords, we identified relevant clinical and observational studies on GLP-1RAs for alcohol use disorder, excluding off-topic or non-English works and assessing all studies for eligibility. Results: Out of 1080 records identified, seven studies met the inclusion criteria. The findings from recent clinical trials, large-scale observational studies, and real-world evidence suggest that GLP-1RAs may significantly reduce alcohol consumption, cravings, and alcohol-related hospitalizations. Their central effect on reward processing, coupled with a generally favorable safety profile, supports their potential therapeutic role beyond metabolic disorders. Conclusions: Emerging evidence positions GLP-1RAs as a promising new pharmacologic approach for managing AUD. Ongoing and future research should prioritize larger, longer-duration randomized controlled trials that include diverse populations, with specific attention to treatment motivation, co-occurring psychiatric conditions, and long-term outcomes.

## 1. Introduction

As of 2024, glucagon-like peptide-1 receptor agonists (GLP-1RAs) rank among the top-selling drugs globally and are gaining recognition as potential therapeutic agents for alcohol use disorder (AUD), with growing interest in their roles in modulating alcohol consumption and alcohol craving (Table 1).

AUD is the most common substance use disorder (SUD) worldwide [1]. Current Food and Drug Administration (FDA)-approved pharmacotherapy for AUD includes disulfiram, acamprosate, and naltrexone. However, approximately 50% of patients do not experience optimal outcomes, underscoring the urgent need for more effective anti-craving treatments [2,3].

GLP-1RAs have emerged as promising candidates in addiction medicine due to recent clinical and real-world evidence demonstrating their potential as a novel and effective therapy for AUD [4,5,6]. GLP-1RAs were originally developed for managing type 2 diabetes by enhancing insulin secretion and reducing appetite [7]. Several studies suggest that semaglutide may have a potential benefit for patients with AUD in real-world populations [4,8]. Also, a recent clinical trial showed that exenatide, which crosses the blood–brain barrier [9], significantly reduced heavy drinking days and total alcohol intake in a subgroup of obese patients, as determined by body mass index (BMI) > 30 kg/m2 [4]. GLP-1RAs have shown promising effects on the brain’s reward system, particularly in regulating dopamine signaling, which plays a critical role in addictive behaviors. However, the precise mechanism remains to be elucidated [10].

GLP-1 is a 30-amino-acid peptide produced by the cleavage of proglucagon. It is produced in the neurons in the solitary tract nucleus, pancreatic islet α-cells, and intestinal mucosal L-cells [11]. Dipeptidyl peptidase IV (DPP-4) catalyzes the enzymatic degradation of GLP-1, which results in the loss of its biological efficacy [11]. GLP-1 binds to GLP-1 receptor (GLP-1R), a core member of the G-protein-coupled receptor (GPCR) family, which in turn regulates blood glucose levels and lipid metabolism. GLP-1 is also synthesized in the brain and plays a pivotal role in neuroprotection through the activation of GLP-1 receptor signaling pathways [12]. It augments learning and memory processes in the hippocampus, facilitates neurogenesis, diminishes inflammation and apoptosis, modulates reward behavior, and decreases food consumption [12,13]. The peptide’s half-life has been extended through improved pharmacokinetics, which also extends exposure and duration of action. Currently, GLP-1 receptor agonists are in clinical use for the treatment of type-2 diabetes and obesity [7,14]. In addition, they have therapeutic potential for neurodegenerative diseases [15,16]. Due to its very short plasma half-life (1.5–5 min), GLP-1 has limited therapeutic utility [17]. To overcome its pharmacokinetic limitations, longer-acting GLP-1RAs have been developed that are resistant to DPP-4 degradation and renal clearance.

Several GLP-1RAs have been approved by the United States FDA for the treatment of type 2 diabetes or weight management (Table 2). Because of the poor bioavailability of peptide drugs, most GLP-1 RAs are administered as subcutaneous injections on a daily or weekly basis. Notably, the half-lives of GLP-1RAs and analogs vary depending on the specific formulation and individual differences. Additionally, Tirzepatide is a first-in-class dual incretin receptor agonist that targets both the GLP-1 and GIP (glucose-dependent insulinotropic polypeptide) receptors, which are four-amino-acid peptides synthesized by the K cells of the duodenum and jejunum [18]. Triple agonists (glucagon, GIP, and GLP-1 receptors), like retatrutide [19], have not yet been evaluated in the context of AUD. This review focused on the current clinical trials and real-world evidence with regard to the effects of GLP-1RAs as novel therapeutics for AUD. We also discussed early but encouraging results from clinical trials in AUD, observational and real-world evidence, safety profiles, psychiatric considerations, and future directions leading beyond GLP-1RAs.

This study highlights critical gaps in current AUD treatment, noting the limited efficacy of FDA-approved medications and the unclear mechanisms underlying promising new therapies, such as GLP-1RAs. It provides a novel, interdisciplinary review of clinical and real-world evidence supporting GLP-1RAs for AUD. This review underscores the need for precision psychiatry approaches, including personalized treatment based on multi-omic profiles and advanced tools, like iPSC models and AI. It also calls attention to underexplored dual and triple agonists, advocating for future research that integrates mechanistic rigor with innovative drug repurposing strategies to improve outcomes in AUD.

## 2. Methods

A comprehensive literature search was conducted for this review using the following academic databases, PubMed, Medline, Google Scholar, and Web of Science, accessed on 30 January 2025. The search was limited to English-language articles, with no restrictions on ethnicity or geographical location. Keywords used included ‘GLP-1RA’ and ‘AUD’, ‘alcohol use disorder’, ‘human study’, ‘novel treatment’, ‘GLP-1’, ‘GLP-1 and GIP’, ‘clinical trial’, and ‘real world data’. Studies were excluded if they were off-topic or not published in English. We then carefully reviewed the remaining articles to assess their relevance. Specifically, our inclusion criteria encompassed both clinical trials and observational studies that examined the effects of GLP-1RAs in the context of AUD. In addition, we searched ClinicalTrials.gov, EudraCT, and relevant conference abstracts to identify additional eligible studies and ongoing trials. A narrative review approach was employed to discuss the review findings, as there is a scarcity of human studies on this subject.

## 3. Results

**Figure 1:**
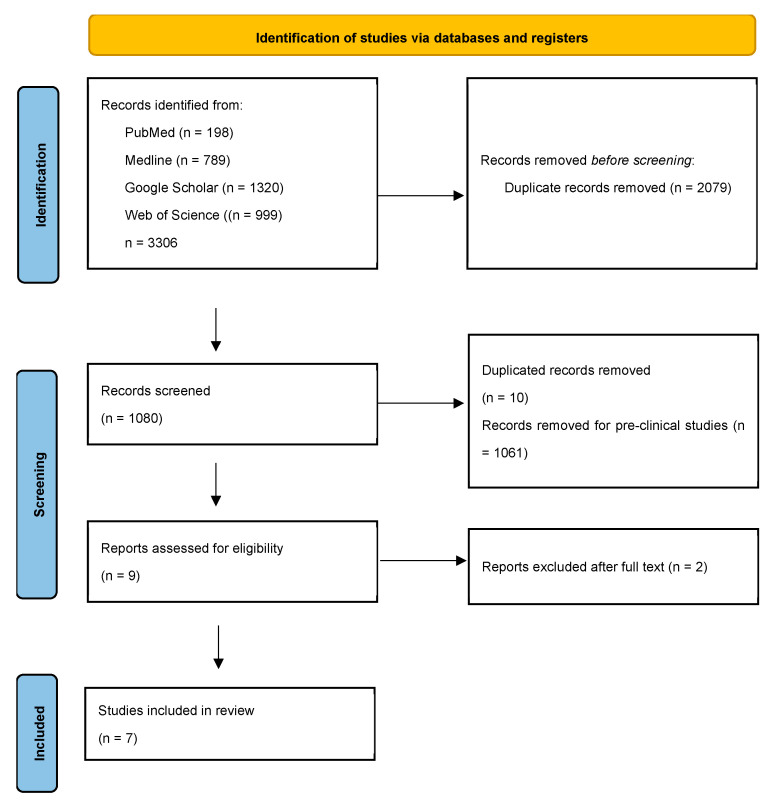
Flow diagram of the review process for GLP-1RA clinical studies in AUD. This PRISMA (preferred reporting items for systematic reviews and meta-analyses) flowchart illustrates the step-by-step selection of articles, including the reasons for study exclusions.

A comprehensive literature review was conducted based on the preferred reporting items for systematic reviews and meta-analysis guidelines (PRISMA), as illustrated in Figure 1. A total of 1080 results were retrieved. Following a thorough screening process, seven studies, including three double-blind placebo control studies and four observational studies, were deemed relevant and subsequently included in this review (Table 3). These selected human clinical studies assess the effectiveness of GLP-1RAs in the human population, focusing on their associations with alcohol use, brain activity, alcohol cravings, and cognitive function. Beyond clinical trials, this review also incorporates real-world data analyses and large-scale observational studies (Table 3).

### 3.1. Clinical Trials: Early but Encouraging Results

The therapeutic potential of GLP-1RAs in AUD was first suggested in a cross-sectional study investigating liraglutide in patients with type 2 diabetes, where reduced alcohol intake was observed as a secondary finding [20]. In 2022, Klausen et al. published the first randomized clinical trial (RCT) to investigate the effects of GLP-1RAs on alcohol consumption, brain function, and alcohol craving in patients with AUD [4] (Table 3). Specifically, Klausen et al. evaluated the effects of exenatide in treatment-seeking individuals with AUD. Although exenatide did not significantly reduce heavy drinking days compared to placebo overall, a subgroup with BMI >30 kg/m^2^ experienced notable reductions in both heavy drinking days and total alcohol intake over the past 30 days [4]. In addition, the neuroimaging study revealed that the exenatide group exhibited reduced alcohol cue reactivity in reward- and addiction-related brain regions, especially the ventral and dorsal striatum. Whole-brain fMRI analyses further demonstrated decreased activation in the left caudate, septal area, and right frontal cortex after 26 weeks of exenatide treatment. These findings suggest that exenatide may decrease the brain’s reward response to alcohol-related cues. Moreover, single-photon emission computed tomography (SPECT) scans showed significantly lower dopamine transporter (DAT) availability in reward-processing areas among those receiving exenatide compared to the placebo. However, no significant differences were observed in subjective alcohol craving or cognitive performance between groups [4].

Subsequently, Probst et al. conducted a randomized controlled trial to investigate the effects of dulaglutide on alcohol consumption during smoking cessation [21] (Table 3). The study reported a 29% reduction in alcohol use compared to the placebo [21]. Importantly, these benefits occurred regardless of the smoking status, indicating that GLP-1RAs may have independent effects on alcohol use. It should be noted that this trial was initially designed to study the effect of dulaglutide on smoking cessation. Thus, the study participants did not, per se, suffer from AUD, and the subgroup of heavy drinkers was too small to provide conclusive evidence [21].

More recently, Hendershot et al. published a phase 2 trial in 2025, evaluating semaglutide in non-treatment-seeking patients [22]. This study found that low-dose semaglutide significantly reduced alcohol cravings, the number of drinks per drinking day, and heavy drinking episodes, with medium to large effect sizes. However, it did not impact the number of drinking days or average drinks per day. These mixed but encouraging results highlight the complexity of treating AUD and the need to tailor interventions to individual patterns of alcohol use and motivation for treatment [22]. Notably, participants in both Probst and Hendershot’s studies were not seeking treatment for AUD [21,22], underscoring the potential utility of GLP-1RAs in broader, real-world clinical contexts. The vast majority of individuals (~90%) with AUD do not seek formal treatment [23]. The efficacy of these medications in non-treatment-seeking populations suggests that they may still provide benefits when prescribed for other conditions [24].

### 3.2. Real-World and Observational Evidence

Beyond clinical trials, observational studies using electronic health records and national registries have provided real-world evidence supporting the therapeutic potential of GLP-1RAs for AUD. Wium-Andersen et al. examined whether the use of GLP-1RA was associated with a decreased risk of alcohol-related events by utilizing data from nationwide registers in the Danish population [25]. Alcohol-related events were defined as (1) hospital contacts with a main diagnosis of AUD (international classification of diseases [ICD]-10 code DF10) in the Danish National Patient Registry, (2) registered treatments for alcoholism in the National Registry of Alcohol Treatment, or (3) the purchase of the benzodiazepine chlordiazepoxide (ATC code N05BA02), which is used for alcohol withdrawal syndrome or the purchase of a medication against alcohol dependence (ATC code N07BB), registered in the Danish National Prescription Registry. GLP-1 receptor agonist users (*n* = 38,544) and dipeptidyl peptidase-4 (DDP-4) inhibitor users (*n* = 49,222) were included in the analysis. This study indicates that, after controlling for covariates, using GLP-1 receptor agonists was linked to a lower risk of a subsequent alcohol-related event than using DPP-4 inhibitors. It should be highlighted that starting GLP-1 receptor agonist therapy was linked to a decreased risk of an alcohol-related event when compared to the time without treatment; however, this association was only seen in the first three months of treatment. Nonetheless, this study concluded that GLP-1RAs did not appear to be viable alternatives to existing treatments for AUD (Table 3) [25].

In the United States, Qeadan et al. analyzed data from over 817,000 individuals with a documented history of AUD from the Oracle Cerner Real-World Data. This study discovered that prescriptions for gastric inhibitory polypeptide (GIP) receptor agonists (tirzepatide; dual agonist for GLP1 and GIP) and GLP-1RAs (albiglutide, dulaglutide, exenatide, liraglutide, lixisenatide, semaglutide) were linked to a 50% decrease in alcohol intoxication events compared to those without a prescription for GIP/GLP-1 RA. Specifically, subjects with AUD who had a prescription for GIP/GLP-1 RA had an incident alcohol intoxication rate that was 49%, 42%, and 42% lower, respectively, when stratified by type 2 diabetes, obesity, and type 2 diabetes and obesity (Table 3) [26].

Wang et al. conducted a large retrospective cohort study using electronic health records from the TriNetX Platform. Of the 83,825 obese patients without a prior diagnosis of AUD, 45,797 received their first prescription for semaglutide and 38,028 received non-GLP-1RA anti-obesity drugs, such as topiramate or naltrexone, between June 2021 and December 2022. Following propensity score matching, the two cohorts were balanced (n = 26,566 in each group, mean age 51.2 years, 65.9% women, 15.8% black, 66.6% white, 6.5% Hispanic). Matched cohorts were followed for one year after the index event. In contrast to non-GLP-1RA anti-obesity drugs, such as topiramate or naltrexone, semaglutide significantly reduced the risk of incident and AUD relapse in patients with obesity or type 2 diabetes (Table 3) [8].

More recently, Lähteenvuo et al. analyzed Swedish nationwide registries to determine whether GLP-1RA use decreased the risk of hospitalization for AUD [4]. Among 227,868 individuals with AUD, semaglutide and liraglutide were associated with the lowest risks of hospitalization for both AUD and other SUDs. In contrast, other GLP-1RAs did not show similar effects. Surprisingly, except for naltrexone, FDA-approved AUD medications (acamprosate and disulfiram) were not associated with reduced hospitalization risk. Additionally, these medications were linked to a small but significant increase in suicide attempt risk (adjusted hazard ratio (aHR), 1.15, 95% CI, 1.08–1.22), whereas GLP-1RAs, including semaglutide, were not (semaglutide: aHR, 0.55, 95% CI, 0.23–1.30) (Table 3) [27]. These findings should be interpreted cautiously due to differing comparator groups and potential confounding [27].

While these observational studies cannot establish causality, their consistency across diverse populations and study designs strengthens the case for continued investigation of GLP-1RAs as therapeutic agents for AUD. GLP-1RAs are noteworthy as important candidates for upcoming randomized trials in AUD, as the epidemiological evidence in the metabolic and psychiatric domains has come together.

### 3.3. Safety and Considerations

The most reported side effects are gastrointestinal in nature—primarily nausea, vomiting, and diarrhea. These symptoms are generally dose-dependent and temporary but can lead to dehydration and, in rare instances, pre-renal acute kidney injury [28]. Despite the therapeutic potential of GLP-1RAs, concerns have been raised regarding potential neuropsychiatric side effects, including depressive symptoms and suicidality. Klausen et al. reported a case who died from suicide two months after discontinuing semaglutide; however, no causal link was addressed [4]. Similarly, several observational studies have also raised concerns about a potential link between GLP-1RAs and the increased risk of depressive symptoms and suicidality [29,30]. However, more recent evidence counters these concerns. Specifically, a large nationwide cohort study conducted in France found no short-term increase in suicide risk among users of GLP-1RAs [31]. Similarly, a meta-analysis of 27 randomized controlled trials reported no significant association between GLP-1RA use and suicidality [32]. Additionally, Lähteenvuo et al. reported that semaglutide was not associated with increased suicide risk, whereas FDA-approved AUD medications were linked to a modestly increased risk [27]. Additional studies have, likewise, found no clear association between GLP-1RA use and suicidality [33,34,35,36,37], further supporting the overall neuropsychiatric safety of this drug class.

## 4. Discussion and Future Perspectives

### 4.1. GLP-1RAs in AUD: Efficacy of GLP-1RAs in AUD and the Role of Obesity

Clinical trials investigating the role of GLP-1RAs in AUD are still in the early stages, but initial findings are encouraging. GLP-1RAs are thought to influence motivation and reward-related behaviors primarily by modulating the brain’s reward system, particularly dopamine signaling pathways in the mesolimbic circuit [38,39,40]. GLP-1 receptors are expressed in key regions involved in reward processing, including the nucleus accumbens and ventral tegmental area. The activation of these receptors has been shown to reduce drug-seeking behaviors [41,42]. Human neuroimaging studies, such as those by Klausen et al., confirmed these findings by demonstrating decreased activation in alcohol cue-reactive brain regions following exenatide administration [4]. Despite no significant reduction in heavy drinking days in patients with AUD overall, when patients were stratified by obesity status, as determined by BMI > 30, significant reductions in heavy drinking days and monthly alcohol intake were observed in patients with BMI > 30. Although the mechanisms underlying this differential response remain unclear, prior research has shown that pharmacokinetic and pharmacodynamic responses to GLP-1RAs vary between lean and obese individuals [43]. Specifically, the administration of intravenous exenatide resulted in an 18.5-fold increase in insulin secretion in lean individuals, as opposed to an 8.8-fold increase in obese individuals [43]. Exenatide, however, dramatically reduced the fMRI signal in the frontal cortex, insula, hippocampus, and amygdala in obese people in response to food pictures but had no effect on the brain fMRI signal in lean participants [43], indicating that the metabolic phenotype may modulate responses of the central nervous system.

### 4.2. Observational Evidence and Real-World Relevance: GLP-1RA Use and Alcohol-Related Outcomes

Observational and real-world studies using large datasets and national registries provide compelling evidence of the association between GLP-1RA use and reduced alcohol-related outcomes. Studies from Denmark, the United States, and Sweden consistently found lower rates of alcohol-related hospital visits, intoxication events, and AUD recurrence among individuals taking GLP-1RAs compared to those on other anti-diabetic or anti-obesity medications (Table 3). The convergence of findings across diverse populations and methodologies strengthens the case for a real therapeutic signal and suggests that these medications may exert beneficial effects across a range of real-world clinical contexts, including patients with comorbid conditions, i.e., obesity and type 2 diabetes. While observational studies are valuable for detecting real-world patterns, they cannot establish causality and may be subject to confounding.

### 4.3. Safety Considerations: Neuropsychiatric Risks, Comorbidity Gaps, and Research Limitations

GLP-1RAs are generally well-tolerated, with gastrointestinal symptoms being the most common adverse events [42]. However, concerns have been raised about potential neuropsychiatric side effects, particularly suicidality. Although isolated case reports and some observational studies suggest a possible link [42,44], the majority of evidence, including large cohort studies and meta-analyses, has not demonstrated a significant increase in suicide risk [29,30]. In contrast, Wang et al. reported that semaglutide may reduce the risk of recurrence suicidal ideation in patients with overweight or obesity when compared to patients treated with non-GLP-1RA anti-obesity medication (HR: 0.44, 96% CI: 0.32–0.60, *n* = 865 each group after propensity score matching) [8]. Some data suggest that GLP-1RAs may pose a lower suicide risk compared to traditional AUD medications, a finding that warrants further investigation [34]. In a retrospective study conducted using the US Department of Veterans Affairs databases (*n* = 1,955,135), Xie et al. found that the use of GLP-1RA was associated with a reduced risk of a series of SUDs, including AUD, opioid use disorder, cannabis use disorder, and stimulant use disorder [45]. Additionally, the risk of suicidal ideation, attempt, or intentional self-harm was lower in patients with diabetes who were taking GLP-1RA compared to those who were taking other anti-diabetes medications, such as sulfonylureas, DPP-4 inhibitors, or sodium–glucose cotransporter-2 inhibitors [45]. Moreover, a recent meta-analysis study showed that GLP-1RAs induced significant reductions in the depression rating scales compared to control treatments [46]. These studies added to the body of evidence on the potential benefits of GLP-1RAs in the treatment of neuropsychiatric disorders. They also stress the need for more investigation into the biology and effectiveness of GLP-1RAs as a primary or adjuvant treatment for the treatment of SUDs, psychotic disorders, and depressive disorders. 

To date, three double-blind, randomized, placebo-controlled clinical trials have investigated the use of GLP-1RAs in AUD (Table 3). Notably, these studies have small sample sizes and short follow-up durations. Furthermore, the heterogeneity in alcohol use patterns, treatment-seeking behavior, and comorbidities among patients complicate efforts to draw broad conclusions about efficacy. To evaluate the long-term effects of GLP-1RAs on depressive symptoms, the study durations of the included studies listed in Table 3 might not be adequate. The main diagnoses (obesity, type 2 diabetes, or AUD), the GLP-1RA agents and dosages, and the control treatments varied amongst the included studies. Despite these limitations, the available evidence suggests that GLP-1RAs hold therapeutic promise for individuals with AUD.

A major limitation in current GLP-1RA clinical research is the frequent exclusion of individuals with comorbid psychiatric conditions, including co-occurring substance use disorders, depression, or suicidal ideation. Many trials enforce strict exclusion criteria for safety reasons, commonly omitting participants with recent psychiatric hospitalization, activity, a history of suicidal ideation, or a history of suicide attempts. While these precautions are ethically sound, they reduce the generalizability of the findings and may obscure the true risk profile of GLP-1RAs in real-world AUD populations, where psychiatric comorbidities are prevalent. For example, individuals with AUD face elevated risks of both depression and suicidality, yet those most at risk are often underrepresented or entirely excluded from trials [2,47,48]. This is evident in the study by Hendershot et al [22]. (see Table 3), which excluded participants with recent suicidal ideation, a history of suicide attempts, or psychiatric hospitalization within the previous six months. Although large-scale observational studies and meta-analyses to date have not found a significant association between GLP-1RA use and suicidality, existing trials may lack sufficient power or population diversity to detect such risks in individuals with AUD. Therefore, more inclusive and longer-term studies are needed to better assess both the therapeutic potential and safety of GLP-1RAs in this high-risk population.

### 4.4. Toward Precision Psychiatry and Future Directions

The efficacy of GLP-1RAs in treating AUD and metabolic diseases varies across agents [27,49], suggesting a need for individualized treatment strategies. Precision psychiatry is an emerging field that aims to tailor mental health care to individuals. To date, only three medications (disulfiram, acamprosate, and naltrexone) have been approved by the United States FDA for the treatment of AUD in the United States. More than 30 different drugs have been tested in alcohol clinical trials in the last 30 years [50]. Most of those clinical trials, though, either did not show any effect at all or showed effects that were very small [50]. This may be due to the heterogeneity of AUD phenotypes and the critical knowledge gaps that underlie the pathophysiology of AUD and the mechanisms of action of the medications used to treat AUD [51,52]. Although DSM-5, a symptom-based tool, is used to assist in the diagnosis of mental disorders, its accuracy is not questionable. However, psychiatry lacks biological tools to evaluate or predict clinical outcomes using biological and objective measures [53,54]. The promise of precision psychiatry lies in understanding the complex interplay of biological and environmental factors, ultimately leading to personalized diagnosis, treatment, and prevention strategies [55,56].

GLP-1RAs are believed to influence alcohol-related behaviors by modulating the brain’s reward system, particularly dopaminergic signaling in the mesolimbic pathway [57]. To further elucidate the underlying mechanisms responsible for individual variation in drug treatment response, the patient-derived induced pluripotent stem cell (iPSC) model system offers a valuable platform for investigating the cellular and molecular effects of GLP-1RAs in the central nervous system [58,59]. Importantly, these personalized models can recapitulate human brain tissue, which could be a useful tool to determine individual variability in drug response, potentially guiding future drug development and clinical trial design.

The effectiveness of GLP-1RAs varies, and it has been noted that this is due, at least in part, to differences in the molecular formula (Table 2). Consequently, the half-life and PK/PD profiles are different, suggesting a need for individualized treatment strategies [27,49]. Each drug, even within the same drug class, may possess distinct molecular profiles and mechanisms of action, thereby highlighting the necessity of patient-derived in vitro cell models to evaluate drug action in the brain [60,61,62]. Although current in vitro assays and in vivo models designed to discover potential therapeutic targets in psychiatry are useful, there is an urgent need to establish patient-derived model systems to complement studies that use other models for neuropharmacology research [63,64,65]. The patient-derived iPSC model system offers distinct strengths, including (1) working with cells that retain the patients’ unique genetic background, (2) the ability to recapitulate human brain tissue, and (3) the ability to experimentally manipulate live brain-like cells, which is the beauty of the iPSC-based cell model system. Understanding of how GLP-1RAs work at the cellular and molecular levels could potentially identify new drug targets and drug repurposing opportunities for AUD. The patient-derived iPSC cell model could catalyze a paradigm shift that will add more mechanistic rigor to future clinical trials and revolutionize clinical practice for the treatment of AUD. Furthermore, it is also critical to identify central and peripheral biomarkers (e.g., neuroimaging, blood-based markers) that can predict or monitor individual responses to GLP-1RA treatment in AUD.

### 4.5. Beyond GLP-1RAs

With the technology evolving, it is conceivable to uncover novel therapeutic targets for AUD by employing a multi-omics framework [66,67] and advanced systems biology techniques and using patient-derived iPSC model systems and drugs as molecular probes, i.e., GLP-1RAs [3,60,61,62]. These research tools could offer a robust and reliable capability to detect potential pharmacological targets for future investigations.

One of the major challenges that we are currently dealing with is not a lack of data but rather how we use the data that is already available from biobanks, public databases, clinical trials, preclinical studies, and real-world observation studies to generate a testable hypothesis. We should also point out that novel biological system network algorithms, machine learning, and artificial intelligence platforms have shed new light on disease mechanisms and underlying drug mechanisms, addressing critical challenges in big data-oriented biomedically complex systems [68,69,70,71]. These tools have already led to novel discoveries, thus laying the groundwork for testing novel therapeutic agents [62,72,73]. In addition to recommending larger, longer-term clinical trials, we also emphasize the importance of precision psychiatry approaches—such as the identification of predictive biomarkers, the development of patient-derived iPSC models, and the application of AI and multi-omics tools—to address the heterogeneity of AUD and variability in treatment response to GLP-1RAs. These advanced technologies can enhance mechanistic understanding, enable individualized treatment strategies, and guide more targeted clinical trial designs.

Altogether, the current literature indicates that GLP-1RAs represent a promising new direction in the pharmacologic management of AUD. Their central action on reward pathways, combined with robust real-world evidence of reduced alcohol-related harm and a generally favorable safety profile, suggests that GLP-1RAs could have clinical utility beyond metabolic diseases. As these findings continue to evolve, future research should prioritize larger, longer-term RCTs that evaluate GLP-1RAs across diverse patient populations, with careful attention to treatment motivation, psychiatric comorbidities, and long-term outcomes.

## 5. Conclusions

GLP-1 receptors are expressed in critical brain regions that are involved in the processing of rewards. While clinical research on GLP-1RAs in AUD is still in its early stages, the preliminary findings are promising. Real-world evidence suggests that GLP-1 receptor agonists and analogs could be a compelling new avenue for the treatment of AUD. These medications offer a dual mechanism of action that aligns with the multifaceted nature of addiction by targeting both peripheral metabolic and central reward pathways. Although the evidence is emerging, recent clinical trials, large observational studies, and real-world data point to meaningful reductions in alcohol use, alcohol craving, and alcohol-related hospitalization. Going forward, future research is required to identify which subgroups of individuals, such as those with co-occurring obesity or metabolic disorders, may benefit the most. Furthermore, it is critical to enhance understanding of the long-term safety and efficacy of GLP-1RA in diverse populations. Nevertheless, GLP-1RAs may offer a novel and effective therapeutic strategy for AUD.

## Figures and Tables

**Table 1:** Top ten drugs by sales globally in 2024.

Rank	Drug Name Manufacturer	Sales USD (Billions)	Indication(s)	Pharmacological Class
1	Keytruda (Pembrolizumab) Merck	29.5	Various cancers	Anti-PD1 monoclonal antibody
2	Ozempic (Semaglutide) Novo Nordisk	16.1	Type 2 diabetes and weight loss	GLP-1RA
3	Dupixent (Dupilumab) Sanofi/Regeneron	13.5	Severe atopic dermatitis, asthma, and other conditions	Anti-IL4/IL13 monoclonal antibody
4	Eliquis (Apixaban) BMS/Pfizer	13.3	Anticoagulation	Factor Xa inhibitor
5	Biktarvy (Bictegravir/emtricitabine/tenofovir alafenamide) Gilead	12.6	Infectious diseases (HIV)	HIV treatment
6	Darzalex (Daratumumab) J&J	12	Multiple myeloma	Anti-CD38 monoclonal antibody
7	Opdivo (Nivolumab) BMS/Ono Pharma	11.3	Various cancers	Anti-PD1 monoclonal antibody
8	Comirnaty (Tozinameran) Pfizer/BioNTech	10.8	Infectious diseases (COVID-19)	SARS-COVID-19 vaccine
9	Gardasil (Gardasil 9) Merck/CSL	10	Infectious diseases (HPV)	HPV vaccine
10	Skyrizi (Risankizumab-rzaa) AbbVie	9.9	Various autoimmune disorders	Anti-IL23 monoclonal antibody

**Table 2:** FDA-approved GLP-1RAs.

GLP-1RA	Half Life	Molecular Formula	Approval Year	Indication
Exenatide	2–4 h	C_149_H_234_N_40_O_47_S	2005	Type 2 diabetes
Liraglutide	12–13 h	C_172_H_265_N_43_O_51_	2010	Type 2 diabetes
Albiglutide	4–7 days	C_148_H_224_N_40_O_45_	2014	Type 2 diabetes
Dulaglutide	5–6 days	C_2646_H_4044_N_704_O_836_S_18_	2014	Type 2 diabetes
Semaglutide	~7 days	C_187_H_291_N_45_O_59_	2017	Type 2 diabetes
Tirzepatide *	12–13 h	C_225_H_348_N_48_O_68_	2022	Type 2 diabetes

* Tirzepatide is a dual GIP and GLP-1 receptor agonist.

**Table 3:** Summary of clinical trials and real-world data for GLP-1 receptor agonists (GLP-1RAs) in alcohol use disorder (AUD).

Study	Study Design	Participants	Treatment	Control	Outcome Measures	Key Findings
Klausen et al., 2022	DBRCT Single-site 26-week treatment + 6-month follow-up N:127	Treatment-seeking heavy drinkers with AUD	Exenatide 2 mg SC weekly + CBT (N:62)	Placebo injection + CBT (N:65)	Number of heavy drinking days, as determined by TLFB. fMRI alcohol cue reactivity. SPECT-DAT. Alcohol craving, as determined by PACS.	No significant reduction in heavy drinking days overall. In the obese subgroup (BMI > 30), reduced heavy drinking days and monthly alcohol intake. Reduced fMRI alcohol cue reactivity in key reward areas. Reduced SPECT-DAT availability in the striatum at week 26. No significant change in craving despite imaging findings.
Probst et al., 2023	DBRCT Single-site 12-week treatment + 6-month follow-up N:151	Patients in a smoking cessation trial with comorbid AUD	Dulaglutide 1.5 mg SC weekly + varenicline + counseling (N:76)	Placebo injection + varenicline + counseling (N:75)	Alcohol consumption (questionnaire analogous to TLFB).	Participants receiving dulaglutide drank 29% less than participants receiving a placebo. Changes in alcohol use were not correlated with smoking status at week 12.
Hendershot et al., 2025	DBRCT Single-site 9 weeks treatment + 1week follow-up N:48	Non-treatment-seeking individuals with AUD	Semaglutide 0.25 mg, escalating to 1.0 mg SC weekly (N:24)	Placebo injection (N:24)	Grams of alcohol consumed (lab setting). Peak breath alcohol concentration (BAC). Alcohol consumption (TLFB). Weekly alcohol craving (PACS).	Medium to large effect size for alcohol reduction. Decreased drinks per drinking day and craving. Treatment with semaglutide reduced heavy drinking.
Wium-Andersen et al., 2022	Denmark nationwide retrospective cohort study, 2009–2018, with a median 4.1 years follow-up N:87,676	New users of GLP-1RAs or DPP-4 inhibitors	All GLP-1RAs (N:38,454)	DPP4 inhibitors (DPP-4i) (N:49,222)	The association between use of GLP-1 receptor agonists and the risk of subsequent alcohol-related events in Danish adults. The alcohol-related events measured by the following: (1) Hospital contacts with a main diagnosis of AUD in the Danish National Patient Registry, (2) registered treatments for alcoholism in the National Registry of Alcohol Treatment, or (3) purchase of the benzodiazepine chlordiazepoxide, which is used for alcohol withdrawal syndrome or the purchase of a medication against alcohol dependence.	GLP-1 receptor agonist use was associated with a lower risk of a subsequent alcohol-related event compared with DPP-4i use both within the 90 days after initiation and 1 year of follow-up. However, the self-controlled design, which efficiently accounts for unmeasured between-person confounding, demonstrates that the initiation of GLP-1 receptor agonist treatment was also associated with a lower risk of an alcohol-related event compared with the non-treatment period, but only during the first 3 months after treatment. Overall, this study did not support GLP-1 RAs as an effective alternative to the existing treatment of AUD.
Qeadan et al., 2024	USA De-identified electronic health record data from the Oracle Cerner Real-World Data Retrospective cohort study, 2014–2022, with up to 2 years follow-up N:817,309	Patients with AUD	GIP and/or GLP-1 RAs (albiglutide, dulaglutide, exenatide, liraglutide, lixisenatide, semaglutide, tirzepatide) (N:5621)	No GIP/GLP-1 RA prescription (N:811,688)	Alcohol intoxication events.	Treatments with GIP and/or GLP-1 RAs were associated with a 50% reduction in alcohol intoxication events.
Wang et al., 2024	USA De-identified patient electronic health records within the TriNetX Platform Retrospective cohort study, 2017–2022, with up to 3 years follow-up N:83,825	Patients with obesity and/or T2DM	Semaglutide (N:45,797)	Other anti-obesity and anti-diabetic medications: naltrexone and topiramate (N:38,028)	Incident and recurrent AUD.	Semaglutide was associated with a significantly lower risk of incident AUD diagnosis, as compared to naltrexone or topiramate. Semaglutide was associated with a lower risk of recurrent AUD diagnosis, as compared to non-GLP-1RA anti-obesity medications, i.e., naltrexone and topiramate.
Lähteenvuo et al., 2025	Swedish nationwide electronic registries Retrospective cohort study, 2006–2023, with a median 8.8 years follow-up N:227,868	Patients with AUD	Semaglutide, liraglutide, exenatide, dulaglutide (N:6276)	Other AUD medications (N:75,454)	The primary outcome was AUD hospitalization. Secondary outcomes were any substance use disorder (SUD)-related hospitalization, somatic hospitalization, and suicide attempts.	Semaglutide and liraglutide may be effective in the treatment of AUD. Semaglutide and liraglutide lower alcohol-related hospitalization. Semaglutide and liraglutide lowered hospitalization rates due to somatic reasons. Use of GLP-1RAs was not associated with suicide attempts.

Abbreviations: AUD: alcohol use disorder; CBT: cognitive behavioral therapy; DBRCT: Double Blind, Randomized, Placebo-Controlled Clinical Trial; DPP-4: dipeptidyl peptidase-4; fMRI: Functional Magnetic Resonance Imaging; GIP: glucose-dependent insulinotropic polypeptide; GLP-1RA: glucagon-like peptide 1 receptor agonist; PACS: Penn Alcohol Craving Score; SC: subcutaneous; SPECT: single-photon emission computerized tomography; T2DM: type 2 diabetes mellitus; TLFB: Timeline Follow Back; USA: The United States of America.

## Data Availability

No new data were created or analyzed in this study. Data sharing is not applicable to this article.

## References

[1] Danpanichkul P., Duangsonk K., Díaz L.A., Chen V.L., Rangan P., Sukphutanan B., Dutta P., Wanichthanaolan O., Ramadoss V., Sim B. (2025). The burden of alcohol and substance use disorders in adolescents and young adults. Drug Alcohol Depend..

[2] Ho M.F., Zhang C., Wei L., Zhang L., Moon I., Geske J.R., Skime M.K., Choi D., Biernacka J.M., Oesterle T.S. (2022). Genetic variants associated with acamprosate treatment response in alcohol use disorder patients: A multiple omics study. Br. J. Pharmacol..

[3] Ho M.F., Zhang C., Zhang L., Wei L., Zhou Y., Moon I., Geske J.R., Choi D.-S., Biernacka J., Frye M. (2021). TSPAN5 influences serotonin and kynurenine: Pharmacogenomic mechanisms related to alcohol use disorder and acamprosate treatment response. Mol. Psychiatry.

[4] Klausen M.K., Jensen M.E., Møller M., Le Dous N., Jensen A.-M.Ø., Zeeman V.A., Johannsen C.-F., Lee A., Thomsen G.K., Macoveanu J. (2022). Exenatide once weekly for alcohol use disorder investigated in a randomized, placebo-controlled clinical trial. JCI Insight.

[5] Jerlhag E. (2020). Alcohol-mediated behaviours and the gut-brain axis; with focus on glucagon-like peptide-1. Brain Res..

[6] Aranäs C., Edvardsson C.E., Shevchouk O.T., Zhang Q., Witley S., Blid Sköldheden S., Zentveld L., Vallöf D., Tufvesson-Alm M., Jerlhag E. (2023). Semaglutide reduces alcohol intake and relapse-like drinking in male and female rats. eBioMedicine.

[7] Nauck M.A., Quast D.R., Wefers J., Meier J.J. (2021). GLP-1 receptor agonists in the treatment of type 2 diabetes—State-of-the-art. Mol. Metab..

[8] Wang W., Volkow N.D., Berger N.A., Davis P.B., Kaelber D.C., Xu R. (2024). Associations of semaglutide with incidence and recurrence of alcohol use disorder in real-world population. Nat. Commun..

[9] Athauda D., Maclagan K., Skene S.S., Bajwa-Joseph M., Letchford D., Chowdhury K., Hibbert S., Budnik N., Zampedri L., Dickson J. (2017). Exenatide once weekly versus placebo in Parkinson’s disease: A randomised, double-blind, placebo-controlled trial. Lancet.

[10] Klausen M.K., Thomsen M., Wortwein G., Fink-Jensen A. (2022). The role of glucagon-like peptide 1 (GLP-1) in addictive disorders. Br. J. Pharmacol..

[11] Zheng Z., Zong Y., Ma Y., Tian Y., Pang Y., Zhang C., Gao J. (2024). Glucagon-like peptide-1 receptor: Mechanisms and advances in therapy. Signal Transduct. Target. Ther..

[12] Diz-Chaves Y., Herrera-Pérez S., González-Matías L.C., Mallo F., Litwack G. (2022). Chapter Fifteen—Effects of Glucagon-like peptide 1 (GLP-1) analogs in the hippocampus. Vitamins and Hormones.

[13] Chen B., Yu X., Horvath-Diano C., Ortuño M.J., Tschöp M.H., Jastreboff A.M., Schneeberger M. (2024). GLP-1 programs the neurovascular landscape. Cell Metab..

[14] Drucker D.J. (2024). Efficacy and Safety of GLP-1 Medicines for Type 2 Diabetes and Obesity. Diabetes Care.

[15] Zhou Z.D., Yi L., Popławska-Domaszewicz K., Chaudhuri K.R., Jankovic J., Tan E.K. (2025). Glucagon-like peptide-1 receptor agonists in neurodegenerative diseases: Promises and challenges. Pharmacol. Res..

[16] Siddeeque N., Hussein M.H., Abdelmaksoud A., Bishop J., Attia A.S., Elshazli R.M., Fawzy M.S., Toraih E.A. (2024). Neuroprotective effects of GLP-1 receptor agonists in neurodegenerative Disorders: A Large-Scale Propensity-Matched cohort study. Int. Immunopharmacol..

[17] Hui H., Farilla L., Merkel P., Perfetti R. (2002). The short half-life of glucagon-like peptide-1 in plasma does not reflect its long-lasting beneficial effects. Eur. J. Endocrinol..

[18] Jastreboff A.M., Aronne L.J., Ahmad N.N., Wharton S., Connery L., Alves B., Kiyosue A., Zhang S., Liu B., Bunck M.C. (2022). Tirzepatide Once Weekly for the Treatment of Obesity. New Engl. J. Med..

[19] Jastreboff A.M., Kaplan L.M., Frías J.P., Wu Q., Du Y., Gurbuz S., Coskun T., Haupt A., Milicevic Z., Hartman M.L. (2023). Triple–Hormone-Receptor Agonist Retatrutide for Obesity—A Phase 2 Trial. N. Engl. J. Med..

[20] Kalra S. (2011). Change in Alcohol Consumption Following Liraglutide Initiation: A Real-Life Experience. Proceedings of the 71st American Diabetes Association Annual Meeting 2011.

[21] Probst L., Monnerat S., Vogt D.R., Lengsfeld S., Burkard T., Meienberg A., Bathelt C., Christ-Crain M., Winzeler B. (2023). Effects of dulaglutide on alcohol consumption during smoking cessation. JCI Insight.

[22] Hendershot C.S., Bremmer M.P., Paladino M.B., Kostantinis G., Gilmore T.A., Sullivan N.R., Tow A.C., Dermody S.S., Prince M.A., Jordan R. (2025). Once-Weekly Semaglutide in Adults with Alcohol Use Disorder: A Randomized Clinical Trial. JAMA Psychiatry.

[23] Degenhardt L., Glantz M., Evans-Lacko S., Sadikova E., Sampson N., Thornicroft G., Aguilar-Gaxiola S., Al-Hamzawi A., Alonso J., Helena Andrade L. (2017). Estimating treatment coverage for people with substance use disorders: An analysis of data from the World Mental Health Surveys. World Psychiatry.

[24] Koob G.F. (2024). Alcohol Use Disorder Treatment: Problems and Solutions. Annu. Rev. Pharmacol. Toxicol..

[25] Wium-Andersen I.K., Wium-Andersen M.K., Fink-Jensen A., Rungby J., Jørgensen M.B., Osler M. (2022). Use of GLP-1 receptor agonists and subsequent risk of alcohol-related events. A nationwide register-based cohort and self-controlled case series study. Basic Clin. Pharmacol. Toxicol..

[26] Qeadan F., McCunn A., Tingey B. (2024). The association between glucose-dependent insulinotropic polypeptide and/or glucagon-like peptide-1 receptor agonist prescriptions and substance-related outcomes in patients with opioid and alcohol use disorders: A real-world data analysis. Addiction.

[27] Lahteenvuo M., Tiihonen J., Solismaa A., Tanskanen A., Mittendorfer-Rutz E., Taipale H. (2025). Repurposing Semaglutide and Liraglutide for Alcohol Use Disorder. JAMA Psychiatry.

[28] Dong S., Sun C. (2022). Can glucagon-like peptide-1 receptor agonists cause acute kidney injury? An analytical study based on post-marketing approval pharmacovigilance data. Front. Endocrinol..

[29] Schoretsanitis G., Weiler S., Barbui C., Raschi E., Gastaldon C. (2024). Disproportionality Analysis From World Health Organization Data on Semaglutide, Liraglutide, and Suicidality. JAMA Netw. Open.

[30] Kornelius E., Huang J.Y., Lo S.C., Huang C.N., Yang Y.S. (2024). The risk of depression, anxiety, and suicidal behavior in patients with obesity on glucagon like peptide-1 receptor agonist therapy. Sci. Rep..

[31] Bezin J., Benard-Laribiere A., Hucteau E., Tournier M., Montastruc F., Pariente A., Faillie J.L. (2025). Suicide and suicide attempt in users of GLP-1 receptor agonists: A nationwide case-time-control study. EClinicalMedicine.

[32] Ebrahimi P., Batlle J.C., Ayati A., Maqsood M.H., Long C., Tarabanis C., McGowan N., Liebers D.T., Laynor G., Hosseini K. (2025). Suicide and Self-Harm Events with GLP-1 Receptor Agonists in Adults with Diabetes or Obesity: A Systematic Review and Meta-Analysis. JAMA Psychiatry.

[33] Ueda P., Söderling J., Wintzell V., Svanström H., Pazzagli L., Eliasson B., Melbye M., Hviid A., Pasternak B. (2024). GLP-1 Receptor Agonist Use and Risk of Suicide Death. JAMA Intern. Med..

[34] Wang W., Volkow N.D., Berger N.A., Davis P.B., Kaelber D.C., Xu R. (2024). Association of semaglutide with risk of suicidal ideation in a real-world cohort. Nat. Med..

[35] Wadden T.A., Brown G.K., Egebjerg C., Frenkel O., Goldman B., Kushner R.F., McGowan B., Overvad M., Fink-Jensen A. (2024). Psychiatric Safety of Semaglutide for Weight Management in People Without Known Major Psychopathology: Post Hoc Analysis of the STEP 1, 2, 3, and 5 Trials. JAMA Intern. Med..

[36] Kim T.H., Lee K., Park S., Cho H., Park J., Jo H., Son Y., Kim S., Kang J., Smith L. (2024). Association between glucagon-like peptide-1 receptor agonists and risk of suicidality: A comprehensive analysis of the global pharmacovigilance database. Diabetes Obes. Metab..

[37] McIntyre R.S., Mansur R.B., Rosenblat J.D., Kwan A.T.H. (2024). The association between glucagon-like peptide-1 receptor agonists (GLP-1 RAs) and suicidality: Reports to the Food and Drug Administration Adverse Event Reporting System (FAERS). Expert Opin. Drug Saf..

[38] Pannacciulli N., Le D.S., Salbe A.D., Chen K., Reiman E.M., Tataranni P.A., Krakoff J. (2007). Postprandial glucagon-like peptide-1 (GLP-1) response is positively associated with changes in neuronal activity of brain areas implicated in satiety and food intake regulation in humans. Neuroimage.

[39] Gabery S., Salinas C.G., Paulsen S.J., Ahnfelt-Rønne J., Alanentalo T., Baquero A.F., Buckley S.T., Farkas E., Fekete C., Frederiksen K.S. (2021). Semaglutide lowers body weight in rodents via distributed neural pathways. JCI Insight.

[40] Kabahizi A., Wallace B., Lieu L., Chau D., Dong Y., Hwang E.-S., Williams K.W. (2022). Glucagon-like peptide-1 (GLP-1) signalling in the brain: From neural circuits and metabolism to therapeutics. Br. J. Pharmacol..

[41] Merchenthaler I., Lane M., Shughrue P. (1999). Distribution of pre-pro-glucagon and glucagon-like peptide-1 receptor messenger RNAs in the rat central nervous system. J. Comp. Neurol..

[42] Muscogiuri G., DeFronzo R.A., Gastaldelli A., Holst J.J. (2017). Glucagon-like Peptide-1 and the Central/Peripheral Nervous System: Crosstalk in Diabetes. Trends Endocrinol. Metab..

[43] Eldor R., Daniele G., Huerta C., Al-Atrash M., Adams J., DeFronzo R., Duong T., Lancaster J., Zirie M., Jayyousi A. (2016). Discordance Between Central (Brain) and Pancreatic Action of Exenatide in Lean and Obese Subjects. Diabetes Care.

[44] De Giorgi R., Ghenciulescu A., Dziwisz O., Taquet M., Adler A.I., Koychev I., Upthegrove R., Solmi M., McCutcheon R., Pillinger T. (2025). An analysis on the role of glucagon-like peptide-1 receptor agonists in cognitive and mental health disorders. Nat. Ment. Health.

[45] Xie Y., Choi T., Al-Aly Z. (2025). Mapping the effectiveness and risks of GLP-1 receptor agonists. Nat. Med..

[46] Chen X., Zhao P., Wang W., Guo L., Pan Q. (2024). The Antidepressant Effects of GLP-1 Receptor Agonists: A Systematic Review and Meta-Analysis. Am. J. Geriatr. Psychiatry.

[47] Sung Y.K., La Flair L.N., Mojtabai R., Lee L.C., Spivak S., Crum R.M. (2016). The Association of Alcohol Use Disorders with Suicidal Ideation and Suicide Attempts in a Population-Based Sample with Mood Symptoms. Arch. Suicide Res..

[48] Cook S., Osborn D., Mathur R., Forbes H., Parekh R., Maini A., Neves A.L., Gnani S., Beaney T., Walters K. (2024). Is alcohol use disorder associated with higher rates of depression and anxiety among people with new onset type 2 diabetes? A cohort study using linked primary care data in England. BMC Prim. Care.

[49] Madsbad S. (2016). Review of head-to-head comparisons of glucagon-like peptide-1 receptor agonists. Diabetes Obes. Metab..

[50] Litten R.Z., Falk D.E., Ryan M.L., Fertig J., Leggio L. (2020). Five Priority Areas for Improving Medications Development for Alcohol Use Disorder and Promoting Their Routine Use in Clinical Practice. Alcohol. Clin. Exp. Res..

[51] Kwako L.E., Momenan R., Grodin E.N., Litten R.Z., Koob G.F., Goldman D. (2017). Addictions Neuroclinical Assessment: A reverse translational approach. Neuropharmacology.

[52] Litten R.Z., Falk D.E., Ryan M.L., Fertig J.B. (2016). Discovery, Development, and Adoption of Medications to Treat Alcohol Use Disorder: Goals for the Phases of Medications Development. Alcohol. Clin. Exp. Res..

[53] Abo R., Hebbring S., Ji Y., Zhu H., Zeng Z.B., Batzler A., Jenkins G.D., Biernacka J., Snyder K., Drews M. (2012). Merging pharmacometabolomics with pharmacogenomics using ‘1000 Genomes’ single-nucleotide polymorphism imputation: Selective serotonin reuptake inhibitor response pharmacogenomics. Pharmacogenetics Genom..

[54] Roden D.M., Altman R.B., Benowitz N.L., Flockhart D.A., Giacomini K.M., Johnson J.A., Krauss R.M., McLeod H.L., Ratain M.J., Relling M.V. (2006). Pharmacogenomics: Challenges and opportunities. Ann. Intern. Med..

[55] Motsinger-Reif A.A., Jorgenson E., Relling M.V., Kroetz D.L., Weinshilboum R., Cox N.J., Roden D.M. (2013). Genome-wide association studies in pharmacogenomics: Successes and lessons. Pharmacogenetics Genom..

[56] Giacomini K.M., Yee S.W., Ratain M.J., Weinshilboum R.M., Kamatani N., Nakamura Y. (2012). Pharmacogenomics and patient care: One size does not fit all. Sci. Transl. Med..

[57] Subhani M., Dhanda A., King J.A., Warren F.C., Creanor S., Davies M.J., Eldeghaidy S., Bawden S., Gowland P.A., Bataller R. (2024). Association between glucagon-like peptide-1 receptor agonists use and change in alcohol consumption: A systematic review. eClinicalMedicine.

[58] Wu J.C., Garg P., Yoshida Y., Yamanaka S., Gepstein L., Hulot J.S., Knollmann B.C., Schwartz P.J. (2019). Towards Precision Medicine With Human iPSCs for Cardiac Channelopathies. Circ. Res..

[59] Engle S.J., Blaha L., Kleiman R.J. (2018). Best Practices for Translational Disease Modeling Using Human iPSC-Derived Neurons. Neuron.

[60] Ho M.-F., Zhang C., Moon I., Tuncturk M., Coombes B.J., Biernacka J., Skime M., Oesterle T.S., Karpyak V.M., Li H. (2024). Molecular mechanisms involved in alcohol craving, IRF3, and endoplasmic reticulum stress: A multi-omics study. Transl. Psychiatry.

[61] Ho M.-F., Zhang C., Moon I., Biernacka J., Coombes B., Ngo Q., Skillon C., Skime M., Oesterle T., Croarkin P.E. (2023). Epigenetic regulation of GABA catabolism in iPSC-derived neurons: The molecular links between FGF21 and histone methylation. Mol. Metab..

[62] Ho M.-F., Zhang C., Moon I., Zhu X., Coombes B.J., Biernacka J., Skime M., Oesterle T.S., Karpyak V.M., Schmidt K. (2022). Single cell transcriptomics reveals distinct transcriptional responses to oxycodone and buprenorphine by iPSC-derived brain organoids from patients with opioid use disorder. Mol. Psychiatry.

[63] Vadodaria K.C., Ji Y., Skime M., Paquola A.C., Nelson T., Hall-Flavin D., Heard K.J., Fredlender C., Deng Y., Elkins J. (2019). Altered serotonergic circuitry in SSRI-resistant major depressive disorder patient-derived neurons. Mol. Psychiatry.

[64] Jensen K.P., Lieberman R., Kranzler H.R., Gelernter J., Clinton K., Covault J. (2019). Alcohol-responsive genes identified in human iPSC-derived neural cultures. Transl. Psychiatry.

[65] Murai K., Sun G., Ye P., Tian E., Yang S., Cui Q., Sun G., Trinh D., Sun O., Hong T. (2016). The TLX-miR-219 cascade regulates neural stem cell proliferation in neurodevelopment and schizophrenia iPSC model. Nat. Commun..

[66] Laska E.M., Siegel C.E., Lin Z., Bogenschutz M., Marmar C.R. (2020). Gabapentin Enacarbil Extended-Release Versus Placebo: A Likely Responder Reanalysis of a Randomized Clinical Trial. Alcohol. Clin. Exp. Res..

[67] Zillich L., Poisel E., Frank J., Foo J.C., Friske M.M., Streit F., Sirignano L., Heilmann-Heimbach S., Heimbach A., Hoffmann P. (2022). Multi-omics signatures of alcohol use disorder in the dorsal and ventral striatum. Transl. Psychiatry.

[68] da Rocha E.L., Ung C.Y., McGehee C.D., Correia C., Li H. (2016). NetDecoder: A network biology platform that decodes context-specific biological networks and gene activities. Nucleic Acids Res..

[69] Zhang C., Correia C., Weiskittel T.M., Tan S.H., Meng-Lin K., Yu G.T., Yao J., Yeo K.S., Zhu S., Ung C.Y. (2022). A Knowledge-Based Discovery Approach Couples Artificial Neural Networks with Weight Engineering to Uncover Immune-Related Processes Underpinning Clinical Traits of Breast Cancer. Front. Immunol..

[70] Ung C.Y., Ghanat Bari M., Zhang C., Liang J., Correia C., Li H. (2019). Regulostat Inferelator: A novel network biology platform to uncover molecular devices that predetermine cellular response phenotypes. Nucleic Acids Res..

[71] Ghanat Bari M., Ung C.Y., Zhang C., Zhu S., Li H. (2017). Machine Learning-Assisted Network Inference Approach to Identify a New Class of Genes that Coordinate the Functionality of Cancer Networks. Sci. Rep..

[72] Lu J., Baccei A., Lummertz da Rocha E., Guillermier C., McManus S., Finney L.A., Zhang C., Steinhauser M.L., Li H., Lerou P.H. (2018). Single-cell RNA sequencing reveals metallothionein heterogeneity during hESC differentiation to definitive endoderm. Stem Cell Res..

[73] Xing Q.R., El Farran C.A., Gautam P., Chuah Y.S., Warrier T., Toh C.X.D., Kang N.Y., Sugii S., Chang Y.T., Xu J. (2020). Diversification of reprogramming trajectories revealed by parallel single-cell transcriptome and chromatin accessibility sequencing. Sci. Adv..

